# Corticosteroid injection for shoulder pain: single-blind randomized pilot trial in primary care

**DOI:** 10.1186/1745-6215-14-425

**Published:** 2013-12-10

**Authors:** Tim A Holt, David Mant, Andrew Carr, Stephen Gwilym, David Beard, Christy Toms, Ly-Mee Yu, Jonathan Rees

**Affiliations:** 1 Department of Primary Care Health Sciences, Oxford University, Radcliffe Observatory Quarter, Woodstock Rd, Oxford OX2 6GG, England; 2 Nuffield Department of Orthopaedics, Rheumatology and Musculoskeletal Sciences, Oxford University, Oxford, England

**Keywords:** Shoulder pain, Rotator cuff, Tendinopathy, Injection

## Abstract

**Background:**

Shoulder pain is a very common presentation in primary care. Evidence of benefit for subacromial corticosteroid injection is inconclusive and confined largely to studies with short follow-up. We plan a large, definitive, primary-care-based trial to determine efficacy and safety in patients with rotator cuff tendinopathy, and conducted a pilot trial to explore feasibility.

**Methods:**

Six general practitioners (GPs) from Oxfordshire, UK underwent update training in assessing painful shoulders and injecting the subacromial space. Each then recruited patients aged 35 to 74 years from primary care complaining of shoulder pain lasting no more than 6 months. Eligible participants were randomized to receive either methylprednisolone acetate 40 mg with lidocaine 1% (total volume 1 ml), or lidocaine 1% alone (total volume 1 ml), injected into the subacromial space. The participants were blinded to treatment allocation. Feasibility outcomes were rates of recruitment, withdrawal, adherence to the protocol, completeness of follow-up, and success of patient masking. Clinical outcomes were the Oxford Shoulder Score (OSS) at baseline and at 4 and 12 weeks, and responses to three satisfaction questions at 2, 4 and 12 weeks. Outcome data were collected by postal questionnaires.

**Results:**

A total of 40 participants were randomized (80% of the target 50 participants) over 26 weeks giving an overall recruitment rate of 1.5 participants per week. Rates of follow-up were maintained to a high level for the full 12 weeks. Four participants requested a ‘rescue’ corticosteroid injection but no patients withdrew. The trial GPs gave high scores for their confidence that the patient had remained blinded to treatment allocation during the procedure. The OSS at 4 and 12 weeks and the responses to the satisfaction questions are reported.

**Conclusions:**

It is feasible to recruit participants with shoulder pain in the primary care setting for a blinded, randomized trial of corticosteroid injection. Online randomization of participants from the practice is also feasible, and postal questionnaires provide an effective means of gathering outcome data in this area of study. The lessons learned from this pilot will usefully inform the design of a large, definitive efficacy trial in primary care.

**Trial registration:**

Current Clinical Trials ISRCTN82357435

## Background

Shoulder pain is a common complaint with prevalence in the community of around 16% [1]. Shoulder pain can result from a number of underlying disorders including rotator cuff tendinopathy, adhesive capsulitis (‘frozen shoulder’) and osteoarthritis. These conditions may be disabling and carry a significant economic cost [2].

Painful shoulders can be managed in a variety of ways, including analgesia, non-steroidal anti-inflammatory drugs, physiotherapy, and corticosteroid injection [3]. Response to these treatments is unpredictable, and 40% to 50% of patients still have pain at 6 months [4-6]. A significant minority suffers persistent pain and functional impairment [7]. A small proportion requires further investigation and surgical intervention, but this is usually reserved for those failing to respond to conservative therapies.

Corticosteroid injections are widely used in primary care to treat shoulder pain. There is a mixed evidence base to support this treatment and trial results have been inconsistent since the 1980s. Some studies have included all painful shoulder problems, while others investigated patients with a specific underlying diagnosis. A systematic review in 1996 found few studies of adequate methodological quality and evidence for efficacy was scarce [8]. In a subsequent Cochrane review published in 2003 there appeared to be a small benefit of subacromial injection versus placebo for rotator cuff tendinopathy but the authors concluded that the effect may be small and not well maintained [9]. Further research was recommended. A meta-analysis in 2005 suggested benefit of subacromial injection for rotator cuff tendinitis but half of the eight included studies were of low methodological quality [10]. In all cases, the trial injections were delivered by an orthopedic surgeon, rheumatologist, internal medicine specialist or rehabilitation specialist rather than a general practitioner (GP).

The UK primary-care-based SAPPHIRE trial used a factorial design to investigate the effect both of training GPs in treating shoulder problems and of corticosteroid injection versus lidocaine local anesthetic (LA) injection [11]. It found GP training to be cost effective, but the cost effectiveness of corticosteroid over lidocaine was inconclusive [12].

A 2010 systematic review by Coombes *et al*. of both corticosteroid and non-corticosteroid injections for various tendinopathies concluded that corticosteroid injections for lateral epicondylalgia of the elbow (‘tennis elbow’) are limited in their efficacy to a short-term response (4 weeks, range 0 to 12) [13]. For follow-up at intermediate (26 weeks, range 13 to 26) and longer term (52 weeks, range ≥52), outcomes were worse than for no treatment. This review questioned the basis for a very commonly used intervention. The short-term efficacy of corticosteroids for rotator cuff tendinopathy was reported as unclear. It is not possible on current evidence to exclude a clinically meaningful adverse effect of corticosteroid injection on shoulder pain and function following subacromial injection, particularly over intermediate and longer timescales.

A large-scale, primary-care-based trial of corticosteroid injection for rotator cuff tendinopathy is required. To ensure the success, quality and reliability of such a trial, a number of feasibility questions need to be answered. Principally these concern the acceptability of the trial concept and design to patients and healthcare professionals, and the rates of recruitment and adherence to the protocol, all of which are crucial factors to success. A number of studies have investigated corticosteroid injection for shoulder pain and have recruited successfully in the primary care setting. For instance, Hay *et al*. compared corticosteroid injection with physiotherapy [14], and Crawshaw *et al*. compared corticosteroid injection plus exercise with exercise alone [15]. However, we required participants to accept blinded randomization to receive a subacromial injection that might or might not contain corticosteroid and in which the comparator was unlikely to have more than a very short-term effect. The acceptability of this design was therefore important to establish.

Our focus of interest in this pilot study, following the Coombes *et al*. systematic review [13], is rotator cuff tendinopathy rather than adhesive capsulitis. However in general practice the distinction between these conditions is not always obvious and it is known that GPs often adopt a pragmatic approach using non-specific ‘shoulder pain’ codes rather than specific coded diagnoses in the record [16]. A further aim of this feasibility study was therefore to determine how easily these two conditions could be distinguished by general practitioners in the trial screening setting.

## Methods

### Design

This is a single-blind, randomized controlled pilot trial in primary care. General practitioners working at six practices in Oxfordshire, UK were recruited and undertook update training by an orthopedic shoulder surgeon in assessing painful shoulders and injecting the subacromial space. Patients presenting with shoulder pain to any clinician in these general practices were asked if they would be willing to participate.

### Participants

Adult patients aged 35 to 74 years with a clinical diagnosis of rotator cuff tendinopathy or adhesive capsulitis.

### Inclusion criteria

Inclusion criteria are: (1) male or female, aged 35 to 74 years; (2) diagnosis of rotator cuff tendinopathy or adhesive capsulitis by a general practitioner trained in shoulder assessment, with duration of symptoms no greater than 6 months; (3) willing to allow his or her usual GP to be notified of participation in the study and to be contacted for further information if an adverse event related to the trial occurs; (4) able to complete follow-up data collection by questionnaire at 2, 4, and 12 weeks; and (5) willing and able to give informed consent for participation in the trial.

### Exclusion criteria

Exclusion criteria are: (1) female participants who are pregnant, lactating or planning pregnancy during the course of the study; (2) participants who have participated in another research study involving an investigational product in the past 12 weeks; (3) those having already received a shoulder injection in the past 12 months; (4) those with other established chronic shoulder disorders (for example, rheumatoid arthritis, other inflammatory polyarthropathies and osteoarthritis); (5) a history of previous shoulder surgery on the affected side; (6) those with evidence of active infection anywhere, including temperature >37.5°C; (7) those currently prescribed anticoagulants; (8) those either currently prescribed, or likely to need during the following 12 months, systemic corticosteroids for any reason; (9) immunocompromised patients; (10) uncontrolled diabetes or uncontrolled hypertension; (11) a diagnosis of heart failure; (12) those considered unable due to cognitive impairment to reliably report outcome measures; (13) any other significant disease or disorder which, in the opinion of the general practitioner, may either put the participants at risk because of participation in the study, or may influence the participant’s ability to participate in the study; and (14) any patient who is allergic to corticosteroids and/or lidocaine.

### Recruitment

GPs interested in participating in the study were identified from practices within the Oxfordshire region. One GP in each of six recruiting practices was identified as the ‘trial GP’. Prior to recruitment of participants, trial GPs underwent study specific training in the assessment of shoulder pain, including the distinction of rotator cuff tendinopathy from adhesive capsulitis and the distinction of these two conditions and other disorders not relevant to this study. They also received training on the injection technique into the subacromial space employed in this trial. Training was led by an academic orthopedic shoulder surgeon from the Nuffield Orthopaedic Centre at Oxford and is based on the British Elbow and Shoulder Society (BESS) Pathway Guideline for Sub-acromial Pain [17]. The trial GPs also received study specific good clinical practice (GCP) training and training in the study procedures (for example, case report form (CRF) completion, safety reporting) from the Primary Care Clinical Trials Unit. The training took less than 1 day to complete.

Potential participants were identified presenting to primary care complaining of shoulder pain. They were given information about the study and if willing to take part in the trial asked to make a 60-minute appointment with the participating trial GP at that GP practice, (not necessarily their usual GP) no earlier than the following day after invitation. This provided the opportunity to read the information and make a decision over participation.

### Screening and baseline assessment

During the 60-minute appointment, a screening form was completed by the trial GP to confirm eligibility. Brief, anonymized demographic information (age, gender, self-declared ethnicity) was collected for all patients screened and blood pressure and temperature measurements were taken. If the person was ineligible, the trial GP discussed clinical management of the shoulder problem with the patient and arranged appropriate care outside the trial. If the participant was eligible, the trial GP then conducted the consent process using the Consent Form. A baseline measure of the OSS was made, and other baseline data collected on a Baseline Assessment Form, including: shoulder symptoms (location, presence of arm pain, neck pain, shoulder stiffness, duration of symptoms, suspected triggers (for example, overuse/strain)); clinical findings, including range of lateral rotation of the glenohumeral joint (limitation might be due to either pain or stiffness); medical history (presence of physical disability, vascular disease, diabetes, hypertension); and concomitant medication (all prescribed medication was recorded on the CRF as well as ‘over-the-counter’ medication for the shoulder problem itself).

### Randomization

The trial GP obtained a randomization allocation online from the Primary Care - Clinical Trials Unit (PC-CTU) at Oxford University. Randomization occurred only after consent to injection on that same day had been obtained. Varying block sizes of 2 or 4 were used with 1:1 allocation to methylprednisolone acetate with lidocaine or lidocaine alone.

### Interventions

The trial injection (methylprednisolone acetate 40 mg with lidocaine 1% in 1 ml, or lidocaine 1% in 1 ml alone) was given according to the randomized allocation by injection into the subacromial space.

The vials were sourced and labeled by Almac Clinical Services Ltd (Almac House, 20 Seagoe Industrial Estate, Craigavon, BT63 5QD, UK) and stored according to the manufactures specifications in the general practice premises. Due to the cloudy appearance of the corticosteroid suspension (compared with the clear appearance of the lidocaine alone) it was not in practice possible to mask the trial GP to the treatment allocation. Every effort was made to ensure that the participants remained masked throughout the procedure. This was made easier as the injection was through a posterior approach. The allocation was not written in the health record and the other GPs in the practice (which in many cases included the ‘usual consulting GP’) were therefore also unaware of the allocation.

### Primary outcome measures

Primary outcome measures are: proportion of screened patients eligible to enter the study, proportion of eligible patients willing to provide consent, rates of recruitment (patients per practice per week), loss to follow-up including withdrawal from the trial, and adherence to the allocated treatment.

### Secondary outcome measures

Secondary outcome measures are Oxford Shoulder Score (OSS) [18] at 4 and 12 weeks following injection, and responses to three satisfaction questions at 2, 4 and 12 weeks post injection: (1) ‘How are the problems related to your shoulder NOW, compared with before your shoulder injection?’ (possible responses: no problems at all now; much better; slightly better; no change; slightly worse; much worse), (2) ‘Overall, how pleased have you been with the result of your shoulder injection?’ (possible responses: very pleased; fairly pleased; not very pleased; very disappointed), and (3) ‘If you could go back in time, would you still choose to have the shoulder injection?’ (possible responses: yes; no; not sure).

The OSS refers to the symptom pattern and shoulder function over the preceding 4 weeks. It was not therefore meaningful to measure this at the 2-week follow-up interval. The OSS has been found to have good sensitivity, validity and responsiveness (ability to detect important changes over time) [19] as well as a high postal return rate [20].

### Outcome data collection

The trial GPs returned screening/eligibility assessment forms and baseline questionnaires, yielding data on recruitment rates and the proportions of screened patients found to be eligible, eligible participants consented, and consented participants randomized. The participants were handed a follow-up questionnaire to complete at 2 weeks following the injection. Subsequent follow-up questionnaires (at 4 weeks and 12 weeks) were sent by post a week before the due date. The participants were reminded by telephone, text or email (according to their stated preference) the day before the assessment was due, if they were willing to receive such reminders. If the form was not received by the research team by 4 days after this due date, a maximum of two non-optional reminders were issued via telephone, text or email. Their wording was as follows: ‘This is a reminder that your follow-up shoulder assessment is due on (date). Please complete the questionnaire and return it to the Research team in the FREEPOST envelope. Thank you’.

Information on other treatments for the shoulder pain (for example, physiotherapy, prescribed or ‘over-the-counter’ analgesia, non-steroidal anti-inflammatory drugs, rescue injection) was also gathered using the questionnaire.

After 12 weeks, the participant’s medical records were reviewed by either the trial GP or the patient’s usual GP, to provide information on other treatments the participant may have received for their shoulder but not mentioned on the questionnaire. Such other treatments might include a ‘rescue’ injection, that is, a definite corticosteroid injection given at the participant’s request before the end of the 12-week study follow-up. Such a treatment represented a protocol deviation but the participant remained in the trial unless they declined follow-up and requested no further contact with the research team.

### Other outcomes

To further inform feasibility we measured the subjective confidence of the trial GP that the participant had remained blind to treatment allocation during the procedure, on a scale of 0% (certain that the allocation had been revealed) to 100% (certain that the participant remained unaware). We gathered feedback from GPs on the adequacy of informational materials and other documentation to support the trial.

### Statistical analysis plan

Descriptive data are presented for all feasibility and clinical outcomes. We derived confidence intervals for difference between trial arms in change from baseline in the OSS at 4 and 12 weeks using analysis of covariance, adjusting for OSS at baseline.

### Ethical approval and consent

The study was approved by NRES Committee South Central, Oxford B, Bristol REC Centre, Ref: 12/SC/0233, EudraCT number: 2012-000147-27.

Consent was obtained from participants prior to randomization for the following: receipt of the trial injection, forwarding of contact details to the research team, willingness to receive up to two reminders in the event of delay in returning outcome data, and examination of medical records by a GP for the 12 weeks following the trial injection.

## Results and discussion

**Figure 1:**
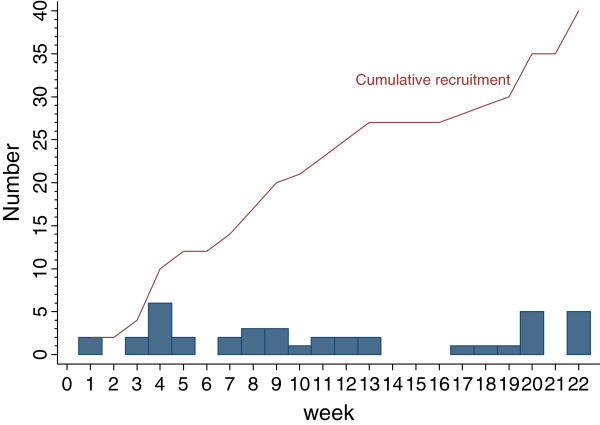
Cumulative recruitment during the 26 weeks of the trial.

**Figure 2:**
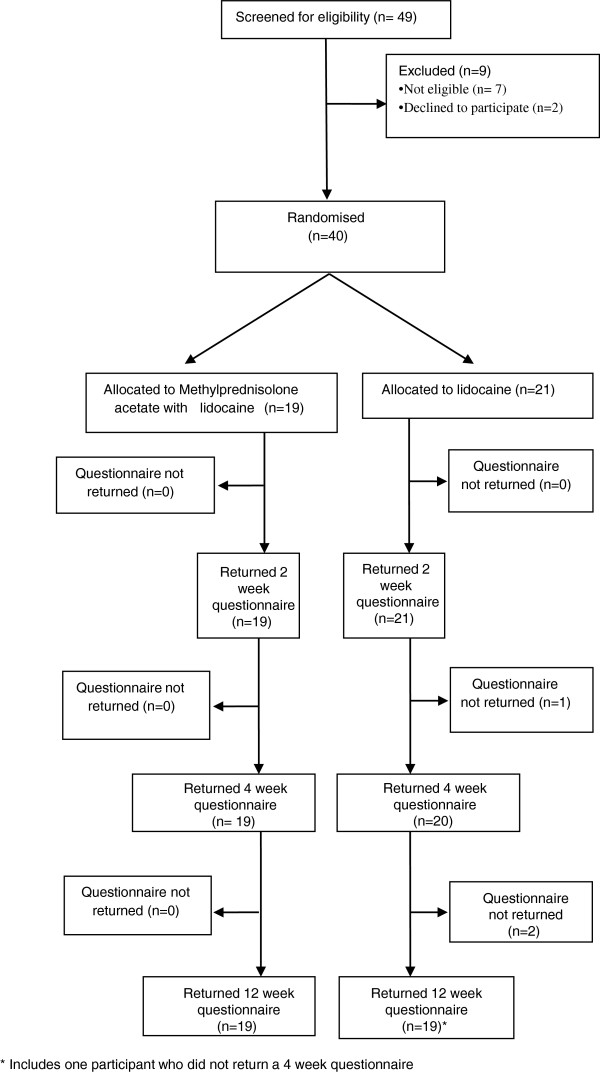
Flow diagram of trial participants.

The pilot ran for 26 weeks during which no serious adverse events were reported. Table 1 gives the recruitment rate by practice from the date of the first site initiation and Figure 1 the cumulative recruitment from the date of first patient recruited. Of 49 screened participants, 42 were deemed eligible, and of these 2 declined to participate (Figure 2). Of the remaining seven, two were found to have shoulder conditions other than rotator cuff tendinopathy or adhesive capsulitis; two had a temperature >37.5°C; one was less than 35 years old; one had a diagnosis of heart failure; and one was taking an oral anticoagulant. All consented participants were randomized and received the trial injection. Recruitment took longer than anticipated but 80% of the planned sample of 50 participants was achieved by study completion. It was clear that trial GPs required the awareness of their GP partners to identify potential participants to be referred into the study. The sixth GP was brought in towards the end of the study to improve recruitment rates and this move proved successful. The background population of all registered patients was 63,663, out of which 31,249 were aged 35 to 74 years.

**Table 1:** Recruitment rates in the six trial practices

**Practice**	**Weeks recruiting**	**Number recruited**	**Recruitment rate (per week)**
1	26	12	0.5
2	26	4	0.15
3	24	8	0.3
4	23	4	0.2
5	24	6	0.25
6	5	6	1.2
Overall	26	40	1.5

There were no difficulties reported with administering the trial injection. GP confidence that the patient had remained unaware of the allocation was reported as 100% in 28/40 cases and 80% to 99% in the remaining 12/40 cases, with no significant difference between the trial arms. While we considered this to be satisfactory, we intend to explore the possibility of blinding the trial GPs in the main trial using opaque syringes. This may in practice be difficult as even a small drop of fluid appearing at the needle tip could reveal the allocation. We minimized awareness of allocation in this pilot by ensuring that no clinician other than the GP administering the injection was aware. The allocation was recorded separately from the patients’ electronic records and was therefore not visible to the wider clinical team. All of our clinical outcomes were patient reported.

Table 2 gives the baseline characteristics, which were similar between the trial arms. Six participants (15% of study sample) had less than 50% range of lateral rotation of the glenohumeral joint compared with the unaffected side, suggesting a clinical diagnosis of adhesive capsulitis. It was unclear at the outset exactly what proportion of participants would be in this subgroup and whether from a practical point of view their identification would be possible, given the known uncertainty associated with primary care diagnosis of shoulder pain. The GPs did not report any difficulty applying this decision rule and the pilot suggests that this subgroup is clinically distinguishable on this basis in this setting. It will therefore be possible to exclude those with adhesive capsulitis at the screening stage from a future larger trial where the main focus of interest is on rotator cuff tendinopathy.

**Table 2:** Baseline characteristics of randomized participants

**Characteristic**	**Methylprednisolone acetate with lidocaine (n = 19)**	**Lidocaine (n = 21)**
Female, n (%)	11 (58%)	15 (71%)
Age at randomization in years, mean (SD)	61.5 (5.8)	56.0 (11.3)
Ethnic group:		
White	19 (100%)	19(90%)
Bangladesh/Indian	0	2 (10%)
Have major medical history	9 (47%)	9 (43%)
Right/left handed:		
Right	18 (95%)	18 (86%)
Left	1 (5%)	3 (14%)
Which shoulder affected:		
Right	13 (68%)	12 (57%)
Left	6 (32%)	9 (43%)
History of trigger for the shoulder pain	5 (26%)	6 (29%)
Current medication for shoulder problem:		
Painkillers	11 (58%)	11 (52%)
Anti-inflammatory medication	12 (63%)	11 (52%)
Physiotherapy	3 (16%)	5 (24%)
Other		
Shoulder stiffness addition to shoulder pain	9 (47%)	12 (57%)
Arm pain addition to shoulder pain	17 (89%)	19 (90%)
Neck pain addition to shoulder pain	9 (47%)	7 (33%)
Other symptoms addition to shoulder pain	6 (32%)	6 (29%)
Duration of any symptom in this shoulder for this episode (weeks), mean (SD)	15.9 (6.1)	10.7 (5.0)
History of shoulder pain	5 (26%)	6 (29%)
Systolic blood pressure, mmHg	130 (14.4)	133 (22.4)
Diastolic blood pressure, mmHg	80 (11.2)	77 (14.7)
Body temperature in °C, mean (SD)	36.7 (0.43)	36.7 (0.44)
Rotation of the glenohumeral joint compared to other side:		
Greater that 50%	17 (89%)	17 (81%)
Less than 50%	2 (11%)	4 (19%)

Table 3 reports the rates of completion of outcome data collection by postal questionnaire, indicating that this is an effective means of gathering such data in this area of study. Postal questionnaires were received by the PC-CTU on average 4.12 days after the due date (±4.11 days) (2 week questionnaire 3.9 days (±2.9); 4-week questionnaire at 4.8 days (±4.2); 12-week questionnaire at 3.7 (±4.9). A total of 38 non-optional reminders were sent to participants. At 2 weeks a total of 6 reminders were sent, 10 reminders were sent for the return of 4-week questionnaires and 22 reminders were required for the return of the 12-week questionnaires. The need for a second reminder also increased with time.

**Table 3:** Rates of completion of outcome data collection

**Baseline**	**2 Weeks**	**4 Weeks**	**12 Weeks**
40%	40%	39%	38%
100%	100%	97.5%	95%

Table 4 presents the adherence to the allocated treatment during the study. One participant in the group allocated to methylprednisolone acetate with lidocaine and three in the lidocaine alone group requested a ‘rescue’ corticosteroid injection during the 12-week follow-up. This result is not significant based on these small numbers, but we recognize the potential for lack of blinding of the trial GP to influence this outcome.

**Table 4:** Adherence to the allocated treatment

**Category**	**Methylprednisolone acetate with lidocaine (n = 19)**	**Lidocaine (n = 21)**	***P *** **value**^**a**^
No. (%) adhering to allocated treatment	18 (95%)	18 (86%)	0.607

^a^Fisher’s exact test.

Table 5 gives the OSS outcomes by trial arm. We have derived confidence intervals for the difference between the arms but recognize that the standard deviation (SD) derived in a pilot study such of this may not be a reliable estimate of the SD in real-world practice or in a larger trial [21]. A rise in the OSS score (indicating improvement) was seen in both arms at 4 and 12 weeks compared to baseline, as might be expected. The rise in the lidocaine alone arm was greater than that of the active injection but the difference was not statistically significant in this pilot study. Nevertheless, this result justifies further investigation through a large definitive trial powered to detect such a difference.

**Table 5:** **Oxford shoulder score**^**a**^

**Stage**	**Methylprednisolone acetate with lidocaine (n = 19)**	**Lidocaine**^**b**^	**Estimated difference in change from baseline (95% CI)**^**c**^
Baseline	26.4 (7.4)	25.4 (9.0)	
4 weeks	30.3 (10.5)	32.7 (8.5)	
12 weeks	30.6 (10.8)	32.7 (8.6)	
Change at 4 weeks from baseline	3.9 (8.5)	6.3 (10.1)	-2.4 (-8.1 to 3.4)
Change at 12 weeks from baseline	4.2 (9.1)	8.2 (11.3)	-2.9 (-9.0 to 3.2)

^a^The higher the score the better.

^b^n = 21 at baseline and 19 from 4 weeks onwards.

^c^Adjusted for baseline score.

**Figure 3:**
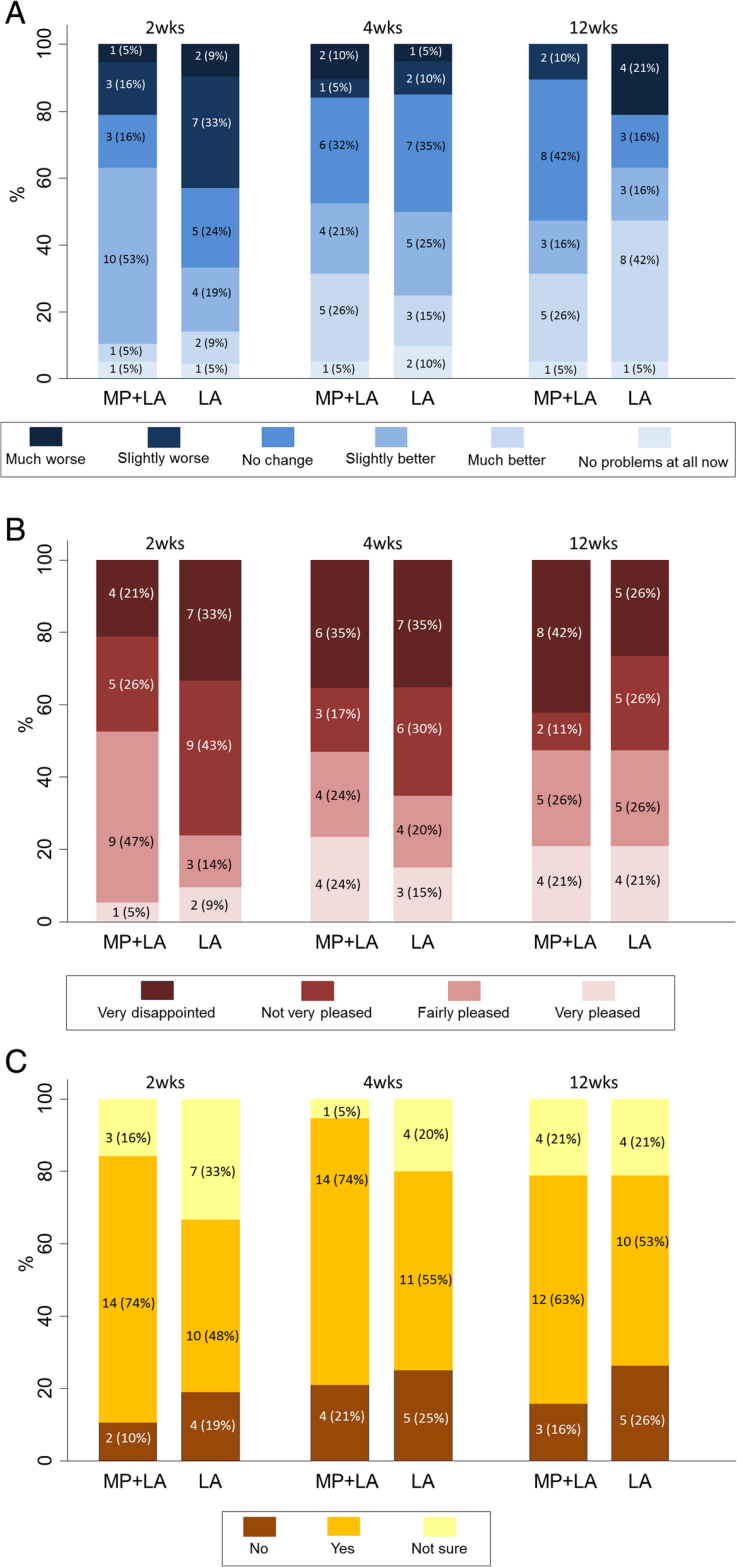
**Responses by trial arm to the three satisfaction questions (MP = methylprednisolone acetate, LA = local anaesthetic). A**: How are the problems with your shoulder NOW compared with before the injection?. **B**: Overall, how pleased have you been with the result of your shoulder injection?. **C**: If you could go back in time, would you still choose to have the shoulder injection?

Figures 3A-C and Table 6 present the response rates to the satisfaction question options in each arm. There is a suggestion of improved satisfaction in the methylprednisolone acetate with lidocaine arm at the 2-week interval compared with lidocaine alone.

**Table 6:** Satisfaction with injection

**Criteria**	**Methylprednisolone acetate with lidocaine (n = 19)**	**Lidocaine (n = 21)**
Responses to first satisfaction question at 2 weeks:		
No problems at all	1 (5%)	1 (5%)
Much better	1 (5%)	2 (10%)
Slightly better	10 (53%)	4 (19%)
No change	3 (16%)	5 (24%)
Slightly worse	3 (16%)	7 (33%)
Much worse	1 (5%)	2 (10%)
Responses to first satisfaction question at 4 weeks:		
No problems at all	1 (5%)	2 (10%)
Much better	5 (26%)	3 (15%)
Slightly better	4 (21%)	5 (25%)
No change	6 (32%)	7 (35%)
Slightly worse	1 (5%)	2 (10%)
Much worse	2 (11%)	1 (5%)
Data missing		1
Responses to first satisfaction question at 12 weeks:		
No problems at all	1 (5%)	1 (5%)
Much better	5 (26%)	8 (42%)
Slightly better	3 (16%)	3 (16%)
No change	8 (42%)	3 (16%)
Slightly worse	2 (11%)	0 (0%)
Much worse	0 (0%)	4 (21%)
Data missing		2
Responses to second satisfaction question at 2 weeks:		
Very pleased	1 (5%)	2 (10%)
Fairly pleased	9 (47%)	3 (14%)
Not very pleased	5 (26%)	9 (43%)
Very disappointed	4 (21%)	7 (33%)
Responses to second satisfaction question at 4 weeks:		
Very pleased	4 (24%)	3 (15%)
Fairly pleased	4 (24%)	4 (20%)
Not very pleased	3 (18%)	6 (30%)
Very disappointed	6 (35%)	7 (35%)
Data missing		1
Responses to second satisfaction question at 12 weeks:		
Very pleased	4 (21%)	4 (21%)
Fairly pleased	5 (26%)	5 (26%)
Not very pleased	2 (11%)	5 (26%)
Very disappointed	8 (42%)	5 (26%)
Data missing		2
Responses to third satisfaction question at 2 weeks		
No	2 (10%)	4 (19%)
Yes	14 (74%)	10 (48%)
Not sure	3 (16%)	7 (33%)
Responses to third satisfaction question at 4 weeks		
No	4 (21%)	5 (25%)
Yes	14 (74%)	11 (55%)
Not sure	1 (5%)	4 (20%)
Data missing		2
Responses to third satisfaction question at 12 weeks		
No	3 (16%)	5 (26%)
Yes	12 (63%)	10 (53%)
Not sure	4 (21%)	4 (21%)
Data missing		2

Finally, Table 7 reports the other treatments received by the trial participants during the study.

**Table 7:** Receipt of other treatments for the shoulder pain during the study

**Treatment/administration**	**Methylprednisolone acetate with lidocaine (n = 19)**	**Lidocaine (n = 21)**
At 2 weeks:		
Painkillers	8/18 (44%)	11/21 (52%)
Anti-inflammatory medication	5/18 (28%)	10/20 (50%)
Physiotherapy	0/17 (0%)	1/20 (5%)
At 4 weeks:		
Painkillers	7/19 (37%)	11/20 (55%)
Anti-inflammatory medication	4/17 (24%)	7/19 (37%)
Physiotherapy	1/16 (6%)	3/19 (16%)
At 12 weeks:		
Painkillers	7/17 (41%)	11/19 (58%)
Anti-inflammatory medication	6/19 (32%)	10/16 (63%)
Physiotherapy	1/18 (6%)	6/18 (33%)

## Conclusions

We succeeded in enrolling 40 participants with shoulder pain in the primary care setting for a blinded, randomized pilot trial of corticosteroid injection. Experience of working with the practices suggested that whole practice involvement in case identification is important to optimize recruitment. Online randomization of participants from the practice supported the single-blinded approach used in this study. Postal questionnaires proved effective for outcome data collection when supported by text or email reminders. The lessons learned from this pilot will usefully inform the design of a large, definitive efficacy trial in primary care. It will be important to optimize recruitment and maintain a high rate of follow-up over 52 weeks in the larger, main trial that we plan. Such a trial is necessary to confirm the benefits of corticosteroid injection, a commonly used treatment for shoulder pain, and to exclude adverse longer-term outcomes.

## Competing interests

The authors declare that they have no competing interests.

## Authors’ contributions

TAH, DM, AC, SG, DB, CT and JR were all involved in the design and conception of the study. CT and TAH led the day-to-day running of the trial. JR trained the trial GPs. L-MY wrote the statistical analysis plan and analyzed the outcome data. All authors contributed to the drafting of the report and approved the final manuscript.
